# Acceptability and Use of Interactive Voice Response Mobile Phone Surveys for Noncommunicable Disease Behavioral Risk Factor Surveillance in Rural Uganda: Qualitative Study

**DOI:** 10.2196/15000

**Published:** 2019-12-03

**Authors:** Charles Ssemugabo, Elizeus Rutebemberwa, Dan Kajungu, George W Pariyo, Adnan A Hyder, Dustin G Gibson

**Affiliations:** 1 Department of Disease Control and Environmental Health Makerere University School of Public Health Makerere University College of Health Science Kampala Uganda; 2 Department of Health Policy, Planning and Management Makerere University School of Public Health Makerere University College of Health Science Kampala Uganda; 3 Iganga Mayuge Health and Demographic Surveillance Site Makerere University Centre for Health and Population Research Kampala Uganda; 4 Department of International Health Johns Hopkins Bloomberg School of Public Health Baltimore, MD United States; 5 Milken Institute School of Public Health George Washington University Washington, DC United States

**Keywords:** mobile phones, interactive voice response, surveillance, behavioral risk factors, noncommunicable diseases, Uganda

## Abstract

**Background:**

There is need for more timely data to inform interventions that address the growing noncommunicable disease (NCD) epidemic. With a global increase in mobile phone ownership, mobile phone surveys can bridge this gap.

**Objective:**

This study aimed to explore the acceptability and use of interactive voice response (IVR) surveys for surveillance of NCD behavioral risk factors in rural Uganda.

**Methods:**

This qualitative study employed user group testing (UGT) with community members. The study was conducted at the Iganga-Mayuge Health and Demographic Surveillance Site (IM-HDSS) in Eastern Uganda. We conducted four UGTs which consisted of different categories of HDSS members: females living in urban areas, males living in urban areas, females living in rural areas, and males living in rural areas. Participants were individually sent an IVR survey, then were brought in for a group discussion using a semistructured guide. Data were analyzed thematically using directed content analysis.

**Results:**

Participants perceived that IVR surveys may be useful in promoting confidentiality, saving costs, and raising awareness on NCD behavioral risk factors. Due to the clarity and delivery of questions in the local language, the IVR survey was perceived as easy to use. Community members suggested scheduling surveys on specific days and sending reminders as ways to improve their use for surveillance. Social issues such as domestic violence and perceptions toward unknown calls, technological factors including poor network connections and inability to use phones, and personal issues such as lack of access to phones and use of multiple networks were identified as barriers to the acceptability and use of mobile phone surveys. However, incentives were reported to motivate people to complete the survey.

**Conclusions:**

Community members reflected on contextual and sociological implications of using mobile phones for surveillance of NCD behavioral risk factors. The opportunities and challenges that affect acceptability and use of IVR surveys should be considered in designing and implementing surveillance programs for NCD risk factors.

## Introduction

The use of mobile phones has a huge potential to promote health in low- and middle-income countries (LMIC) [1-3]. Mobile phones have been used in the surveillance of infectious diseases including malaria, tuberculosis, and influenza-like illness; child malnutrition and maternal health cases; and routine surveillance of various diseases and symptoms [4,5]. In Uganda, mobile phones have been adopted in health programs and used for improving patient care and clinic attendance among HIV/AIDS patients, communication of laboratory results among HIV/AIDS patients, and surveillance [6-9]. However, there is limited literature on use of mobile phones in prevention and control of noncommunicable diseases (NCDs) such as cancer, diabetes, and cardiovascular disease.

NCDs rank fourth among the 10 most common causes of death in Uganda [10]. The increasing burden of NCDs in Uganda and globally is attributed partly to behavioral risk factors including tobacco use, alcohol use, dietary intake, and physical activity [11]. In order to strengthen the health systems response to NCD prevention and control in Uganda, there is a need for frequent data on NCD behavioral risk factors. Surveillance of NCD behavioral risk factors in Uganda has been carried out using the World Health Organization Stepwise Approach to Surveillance (STEPS) NCD risk factor surveillance [12]. However, nationally representative household surveys are resource intensive and therefore are typically only conducted approximately every 5 years [13]. Of note, Uganda last conducted a STEPS survey in 2014 [14]. In order to have more up-to-date data, novel methodologies that can generate data at more frequent intervals need to be explored. Given the high levels of mobile phone ownership and access [15], NCD risk factor surveillance could be explored.

In high income countries, mobile phone surveys have been used to conduct surveillance for NCDs [16-18], but their use in LMICs is limited [19]. Interactive voice response (IVR) is one type of mobile phone survey. In IVR, participants use their mobile phone keypad to answer prerecorded questions. The responses are submitted to a Web database or server to ensure timely data synchronization and analysis. In order to ensure adoption and acceptance of mobile phone surveys for NCD behavioral risk factors in Uganda, there is need to consider the contextual issues in the setting where surveillance will be implemented.

The technology acceptance model (TAM) can help guide the development and adaptation of mobile phone surveys by offering a framework to predict the acceptance and use of information systems and technology by an individual user [20]. It demonstrates that behavioral intention to accept and use the technology is dependent on the perceived usefulness and perceived ease of use; however, these are influenced by external factors within the setting under which the technology is used. Therefore, this paper explores perceptions on the acceptability and use of IVR surveys for surveillance of NCD behavioral risk factors in rural Uganda.

## Methods

### Study Design

This was a qualitative study that employed user group testing (UGT) to inform the development of an IVR survey that can be used to collect NCD behavioral risk factors. Participants were sent an IVR survey to their personal mobile phone and then convened for a group discussion. The UGT approach allowed participants to provide feedback after interacting with the technology [21,22]. Study participants were purposively selected from four categories of people in the study area: females living in urban areas, males living in urban areas, females living in rural areas, and males living in rural areas. One UGT was conducted for each category. This study was designed and conducted in accordance with the Consolidated Criteria for Reporting Qualitative studies (COREQ) [23].

### Study Area and Participants

This study was conducted within the Iganga-Mayuge Health and Demographic Surveillance Site (IM-HDSS). The IM-HDSS is located in two districts, Iganga and Mayuge, in eastern Uganda. The site is about 120 kilometers or 2.5 hours’ drive east of Kampala, the capital city of Uganda, along the Uganda-Kenya highway. A population of 90,000 people resides in 17,000 households with approximately 59% living in rural areas [24]. The site spans 65 villages from 7 subcounties, and it is served by 16 health centers and one hospital. The IM-HDSS conducts 2 regular surveillance rounds of vital statistics per year. Participants were eligible for the study if they were 18 years or older, owned a mobile phone, and had been HDSS residents for at least 6 months.

### Development of the Interactive Voice Response Survey

The IVR survey consisted of questions selected from standardized household surveys such as World Health Organization STEPS, Tobacco Questions for Surveys, and the Behavioral Risk Factor Surveillance System [12,25,26]. Questions that highlighted the indicators in the global monitoring framework for NCDs and covered the four main behavioral risk factors for NCDs (physical activity, alcohol consumption, tobacco use, and dietary intake) were selected [27]. To ensure content validity, the questions were reviewed by mobile health (mHealth), public health, health systems, and HDSS experts.

The selected questions were audio-recorded into Lusoga and uploaded to the Viamo (IVR provider) platform. The IVR survey began with an introduction that included the purpose, duration, benefits, and sponsoring agency. After the survey introduction, participants were asked a series of demographic and NCD risk factor questions where participants use the key pad of their mobile phone to respond (eg, Do you currently smoke tobacco? If yes, press 1. If no, press 3.) Respondent’s data from the IVR survey are stored on a cloud server. The IVR survey was pretested with community members in Kampala prior to the UGTs to ensure that audio files were properly uploaded and that the programming (skip logic and response options) was correct.

### Recruitment

An HDSS staff member with the help of a local community health worker (CHW) approached potential participants at their household and assessed their interest in study participation. The HDSS staff worked with a CHW from the rural area to identify rural UGT participants and an urban-based CHW for urban UGT groups. For those who were interested, the HDSS staff screened community members for study eligibility as previously described. Recruitment for study participation occurred one day prior to the UGT. Recruited participants were told the time, date, and location of the UGT and were asked to bring their mobile phone.

### User Group Testing

The UGTs were carried out in the midmorning at a nearby primary school that was convenient for the study participants. Upon arrival, an HDSS staff member obtained informed consent and collected basic demographic information and mobile phone numbers from each participant. Participants’ mobile phone numbers were then uploaded to the IVR survey platform. In a group setting, the participants were informed about the purpose of the UGT and how to take an IVR survey. Next, all participants were sent the IVR survey to their personal mobile phone by a co-investigator (CS). HDSS staff were available to assist the study participants if they had difficulty answering the survey. In cases where participants did not complete the IVR survey, the IVR survey was resent up to 3 times. This process lasted 30 minutes to 1 hour depending on how fast participants adapted to the exercise.

After participants interacted with the IVR survey, a group discussion was conducted. A semistructured guide developed by the study team was employed to explore perceptions on three thematic areas: (1) acceptability and willingness to using IVR surveys, (2) opportunities and challenges to using IVR surveys, (3) opportunities for improving use of IVR surveys for NCD behavioral risk factor surveillance (Multimedia Appendix 1). The guide was translated to the local language (Lusoga) and back-translated to English for accuracy. The final tool was approved for use by the HDSS site leader. Group discussions were moderated by a public health specialist (CS) and supported by an mHealth specialist (DGG) and two research assistants. The moderator and research assistants were familiar with the IM-HDSS site, spoke the local language (Lusoga), and had experience collecting qualitative data. UGTs were conducted until saturation was reached. All group discussions were audio recorded.

### Data Management and Analysis

The audio recordings of the group discussions were labeled and stored on a secured server. They were transcribed verbatim and translated to English by two experienced research assistants fluent both in English and Lusoga. Two people read the transcripts and developed a codebook. This was done by rereading all the transcripts, assigning meaning units to each response and codes to each meaning unit, and taking note on the emerging subthemes. They combined the descriptive codes and discussed them to produce the code book. Data were entered in ATLAS.ti software version 7.0 (ATLAS.ti Scientific Software Development GmbH) for coding. Qualitative data were analyzed using directed content analysis. We used the TAM as the guiding framework to identify recurrent themes and subthemes by categorizing the codes [20,28]. Emerging themes are presented in the results and supported with quotes. Participant responses in IVR surveys were wirelessly transmitted to a cloud server on the Viamo platform. All IVR surveys taken during the UGT were immediately deleted after the group discussion.

### Ethical Considerations

Ethical approval was obtained from the Makerere University School of Public Health Higher Degrees, Research, and Ethics Committee (protocol 526), Uganda National Council of Science and Technology (registration number SS 4477), and Johns Hopkins Bloomberg School of Public Health. Participation in the study was voluntary, and participants provided written informed consent. Other than the participant’s telephone number, no name or identifying information was used during data collection. Phone numbers in the IVR database were deleted upon completion of the UGT. Data were only assessed by the study team and used only for the purposes of the study. Participants were provided 5000 Ugandan Shillings (US $1.36) for travel reimbursement.

## Results

### Demographic Characteristics

In October 2017, four UGTs with a total of 43 adults selected purposively from the community were carried out. On average, each UGT was composed of 11 people (range 10 to 12 participants). Of the 43 UGT participants, 22 were females (51%), 29 had completed primary school (70%), and the median age was 41 years. The median time to complete the 32-question survey was 18 minutes and 31 seconds.

**Figure 1:**
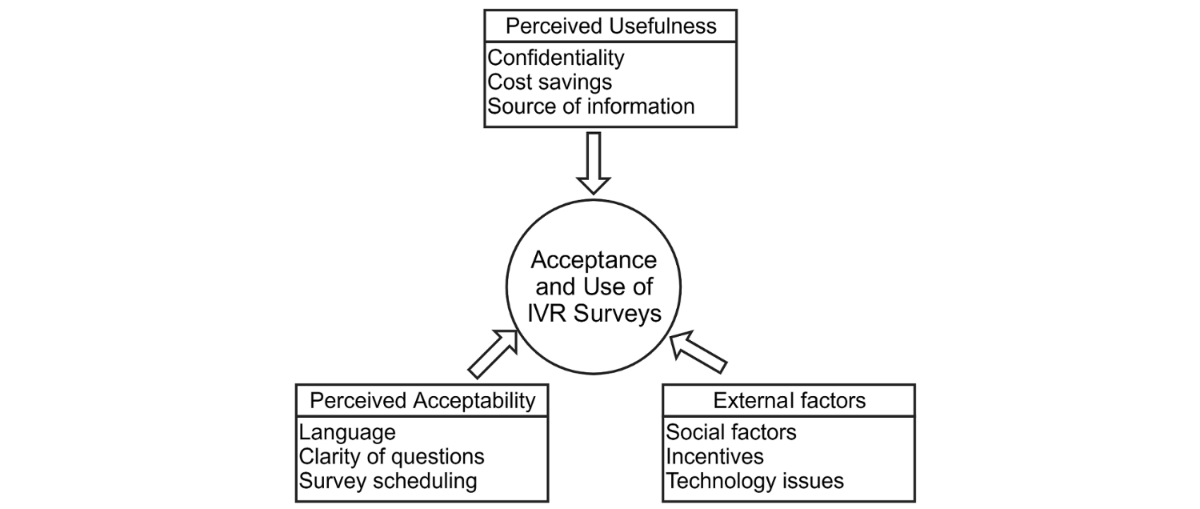
Perceived usefulness and ease of use and external factors that inform use and acceptability of mobile phone surveys in rural Uganda. IVR: interactive voice response.

The findings are organized and presented based on TAM themes: perceived usefulness, perceived ease of use, and external factors (Figure 1). Under perceived usefulness, confidentiality, saving costs, and source of information on NCD risk factors emerged. Language used to administer questions, clarity of questions, and survey scheduling were the themes that emerged under perceived ease of use. External factors included social, technological, personal, and incentives.

### Perceived Usefulness

#### Confidentiality

UGT participants expressed how the IVR-administered questionnaire promotes confidentiality during the interview, commenting that one responds to questions while they are alone. As such, they are likely to give honest and accurate answers.

What I enjoyed most is the fact that the information will remain confidential, a person responds when he/she is alone.

UGT, rural male

#### Saving Costs

Most study participants thought that the IVR-administered questionnaires were saving costs, as one can respond to them at their current location as opposed to traditional face-to-face interviews where they would have to walk back home and meet the interviewer. IVR-administered questionnaires were also viewed as a mechanism of cutting fuel costs used by data enumerators, as the survey is delivered to everyone in a short time.

#### Source of Information on Noncommunicable Disease Risk Factors

Many participants viewed the IVR questionnaire as a source of information on NCD risk factors. They said that the questions highlight their lifestyle choices including diet and work and how they affect their health. The majority said that the surveys reminded them of traditional fruits and vegetables that they had not recently been eating.

The questions asked were concerned with the kind of food that we eat, like fruits such as pawpaw, jackfruit that we had abandoned.

UGT, urban male

### Perceived Ease of Use

#### Language Used to Administer the Interactive Voice Response Survey

All participants perceived the use of local language in the delivery of the IVR-administered questionnaire as one of the motivators to complete the survey. They said that the questions are asked in the language they understand.

It gives you options of selecting the language you want to use to respond to questions, either Lusoga or English. So, the questions are asked in a language that you understand, as such you are able to understand everything very well.

UGT, rural female

#### Clarity of Questions

Almost all UGT participants said that the IVR survey questions were administered in a precise and clear voice, which increased their understanding of questions and hence the quality of data collected. They also mentioned that the questions were administered with clear instructions and sufficient time intervals between questions in a chronological manner.

I like the IVR technology because the voices were coming out clearly as though you are talking to a person physically. I could respond to the questions that were asked very well because I was understanding them.

UGT, rural male

UGT participants viewed introduction to the IVR questionnaire as very important in setting the stage for the respondent and drawing their attention to the subject being surveyed. In fact, they said that the introduction determines the interviewee’s response.

However, some participants expressed challenges in quantifying or estimating the amounts of food, number of steps taken (for physical activity), and number of drinks taken among others. This was attributed to the format in which the questions were designed.

Questions regarding the types of heavy work we do and the time we take doing such work are hard to respond to because some of us don’t have specific work that we do. We do work that comes along. In fact, I can even take a full week without getting heavy work. I may land on an old man and he tells me to go and help him dig in his garden, so answering that question was difficult.

UGT, urban male

#### Survey Scheduling

Participant preferences about scheduling of IVR questionnaire delivery varied with some preferring a specific day and others any day of the week. Participants who preferred a specific day of the week suggested weekends in the morning and others Sunday after lunch. Given the short duration of the survey (18 minutes and 31 seconds), some participants thought they would block off this time to complete the survey.

There should be specific day when the survey is delivered. I have a phone with a weak battery, so this will help me to prepare and charge my phone so that they can call me when I am ready.

UGT, rural female

Community members also thought receiving reminders would increase their chances of completing the IVR questionnaire. Reminders would help them to prepare for the call by charging their phones and ensuring that they are on at the time of the call. However, they had varied opinions on the format in which the reminders should be sent. Some preferred short message service (SMS) messages while others preferred voice messages, especially because many community members cannot read. Another issue was with the period the reminder should be sent prior to the call. Most participants preferred the reminder to be sent one day prior to the call.

### External Factors

#### Social Factors

Participant preference for the voice of the IVR survey was dependent on gender. Females thought that if the survey was recorded in a male voice, their husbands might think they are having an extramarital relationship and thus cause violence in the home. However, some male participants preferred the female voice as a motivator for them to complete the survey.

If the call is recorded in a male voice and the survey comes to a female at night, it may hurt the husband. By the time you explain to him that it is a survey, domestic violence might have occurred already. In such cases, the call should be sent out during the day.

UGT, urban female

Societal constructions were also reported as potential hindrances to acceptance of the IVR survey. Study participants reported that people fear answering unknown calls because they are associated with the Illuminati or perceived evil schemes that can result in death.

Some people fear to receive unknown calls unless you inform them before the survey is delivered that the DSS will call you.

UGT, rural female

#### Incentives

Most participants reported that providing a small incentive would motivate people to complete the IVR interview. The incentives could be provided in either airtime or mobile money and would be given only if the participant completed the survey.

#### Technological Issues

The majority of the UGT participants said that people don’t know how to use mobile phones. In fact, they reported that most people only know how to receive and end calls. Given the survey has questions that might require them to use multiple keypads on their phones, they might not be in position to complete the survey without any training on how the system works. In addition to technology literacy, there may be problems with the respondents’ mobile phone, such as dysfunctional buttons which would prevent them from answering the question.

I was frustrated by the phone. I would understand the instruction but some of the buttons on my phone were not working.

UGT, urban female

Most participants also mentioned network connection as a challenge to attempting and completing the IVR survey.

#### Personal Factors

Lack of access to phones was also reported by most participants as a challenge to using the IVR questionnaire for surveillance of NCD risk factors. They said that some community members, especially in the rural areas, do not have phones while others might have left them charging or in the house at the time the survey is sent. Others don’t have electricity and their phones might not be charged.

Some homes don’t have phones. I don’t know how they will be receiving the IVR survey.

UGT, rural female

It was also reported that some people are on multiple networks yet they have a single network phone. So, they keep switching from one to another. At the time of the call, the known number might be off.

## Discussion

### Principal Findings

Our study provides insights into the acceptability and use of mobile phones for NCD behavioral risk factor surveillance in Uganda. Overall, participants were accepting of the IVR survey and perceived it as a useful tool to monitor a population’s health while also highlighting external factors as potential challenges to its implementation.

The UGTs and group discussions indicated a high level of acceptability given that the survey was narrated in the local language and the questions were clearly asked with appropriate examples as necessary. To improve the likelihood of someone taking an IVR survey, study participants suggested scheduling survey calls on specific days and sending reminders prior to the calls. In Australia, evidence from a randomized controlled trial found that sending text message reminders prior to a mobile phone survey significantly improved the response and completion rates [29]. Although reminders for mobile survey participation have not been assessed in LMICs, there is a large body of evidence supporting their use for other health outcomes [30].

A consensus emerged that IVR surveys are useful in ensuring confidentiality, potentially saving costs, and raising awareness on NCD behavioral risk factors. Whereas delivery of the survey in local language and clarity of questions were perceived to ease the use of the IVR survey, scheduling survey calls on specific days and sending reminders prior to the calls were proposed to ease its use. Community members reported that incentives improve use and acceptability of mobile phone surveys, but social, technological, and personal factors were perceived as barriers to their acceptability and use for NCD behavioral risk factor surveillance.

Privacy and safety of research participants is essential for any research undertaken. Mobile phone surveys using IVR may provide more opportunities to ensure privacy and safety of research participants by removing the human interviewer [31]. In our study, participants perceived that IVR surveys promote confidentiality. This is expected given that face-to-face interviews involve two parties as compared to the IVR questionnaire. Our findings are similar to studies carried out in Uganda and Australia [7,32]. However, studies in Burkina Faso, Uganda, and the United States highlight confidentially as a concern of mHealth research [6,33,34]. Our findings imply that there may be greater acceptance of this new technology as it ensures confidentiality.

Some of the IVR features that facilitated acceptance of the survey were how precise and clearly the questions were asked. Community members mentioned that the clarity of the voice used in the survey’s narration and that instructions were given in a chronological manner prompted them to accept that the survey can be adopted and scaled for use in the HDSS. Given most study participants had not undertaken a survey using the IVR platform before, it is not surprising that clear voices and instructions easily familiarized community members with the technology. Community members also thought that the survey was introduced in a manner that drew the respondent’s attention and eased its completion. Our findings corroborate the observations from another study by members of our group that piloted an IVR survey among university students [35].

Despite the willingness to use the IVR-administered questionnaire, community members felt that some questions that required estimations of their daily activities were difficult to answer. This could be due to lack of proper measurements for the different activities or their failure to make the right estimates or remember details of the activities they were involved with. These findings are also in line with those established during the IVR questionnaire pilot [35]. These findings have implications on the quality of data collected on NCD behavioral risk factors, requiring careful design and selection of questions [36].

The time of day, frequency, and timing of mHealth interventions have been found to influence their acceptance and efficacy elsewhere [30]. In our study, community members shared varied opinions on when the IVR survey should be sent out. While some preferred a specific day of the week (especially weekends), others were happy to receive the survey on any day of the week. This has implications on use and completion of the survey given people have different schedules. It is important that in scheduling the delivery of the IVR questionnaire, community norms and calendar are considered to ensure ease of use of the technology. Giving respondents the ability to schedule when the survey is sent may mitigate this challenge, especially since people are busy at work or their mobile phone may not be charged. It was also evident from our study that reminders may increase community acceptance and use of the IVR-delivered surveys. Most participants preferred voice to SMS reminders because of concerns about illiteracy.

Community members thought that IVR-administered surveys may result in domestic violence between family members if the IVR recording was in a voice opposite from the respondent’s gender. Given that the majority of domestic violence is incurred by women, using a female voice may lessen the likelihood of this event. Participants also thought that societal constructions toward receiving unknown calls would hinder acceptance of the interventions. Communities hold beliefs that some unknown calls are sent by evil spirits that can claim the life of the receiver during the calls. Television, radio, or newspaper campaigns may sensitize communities to inform them about potentially receiving a survey. Moreover, including information on who is sponsoring the survey and offering a phone number for participants to call and verify the survey source may help improve participation. Our findings are similar to others who have explored acceptance of e-interventions such as students’ use of e-learning platforms [37] and the general population’s use of e-banking [38]. The use of financial incentives has been found to increase the behavioral intentions of students, farmers, and the general population to adopt a new technology [39-41]. It was established from our study that financial incentives may increase its acceptance, thereby motivating people to complete the survey. However, this needs to be tested and evaluated as it has implications on sustainability of the surveillance platform.

Use of an IVR-administered questionnaire largely depends on individuals’ ability to use mobile phones and possession of functional phones [42]. Findings from our study hint at potential technology literacy challenges as we found that many community members can only receive calls and switch off their phones. In addition, where individuals can use phones, they are hindered by lack of network connectivity especially in the remote areas. Community members also reported that some of their colleagues do not own mobile phones and others have multiple networks and as such keep switching from one to another. All these technology challenges would prevent individuals from participating in the IVR survey and have been described in other mHealth studies conducted in Uganda. [6]. Like other mHealth interventions, plans to scale-up the use of an IVR-administered questionnaire needs to consider this limitation and may require alternative sampling strategies and survey modalities to reach all potential participants.

### Limitations

The limitations to this study include that perceptions on acceptability and use were explored immediately after subjecting participants to one IVR survey call. This means that their views may have been mainly influenced by the experiences of the interactions during the session. In addition, such a short period of interaction with the innovation might have limited the type of experiences and perceptions that community members shared about acceptability and use of mobile phone surveys during the UGT. In fact, some participants could not respond to or complete the interview due to dysfunctional phones and network challenges. This might have affected the shaping of their opinions toward acceptability and use of mobile phone surveys for NCD risk factor surveillance. Lastly, participants were required to own a mobile phone to participate. Given that phone ownership is not universal, our findings may be biased.

### Conclusion

In conclusion, community members reflected on contextual and sociological implications of using mobile phones for surveillance of NCD behavioral risk factors. Using the TAM, we identified factors under three key themes—perceived usefulness, perceived ease of use, and external factors (technology and personal)—that should be considered in designing and implementing mobile phone surveillance programs for NCD risk factors. Findings from the UGT will be used to refine and adapt the IVR survey and its delivery prior to deployment.

## Appendix

Multimedia Appendix 1 User group discussion guide.

## Abbreviations

CHW: community health worker
COREQ: Consolidated Criteria for Reporting Qualitative Studies
IM-HDSS: Iganga-Mayuge Health and Demographic Surveillance Site
IVR: interactive voice response
LMIC: low- and middle-income countries
mHealth: mobile health
NCD: noncommunicable disease
SMS: short message service
STEPS: Stepwise Approach to Surveillance
TAM: technology acceptance model
UGT: user group testing
