# Supplementary material for: Acceptability and Use of Interactive Voice Response Mobile Phone Surveys for Noncommunicable Disease Behavioral Risk Factor Surveillance in Rural Uganda: Qualitative Study
Source: JMIR Form Res. 2019 Dec 3;3(4):e15000. doi: 10.2196/15000 (PMC6918213; doi:10.2196/15000)
Supplement: Multimedia Appendix 1 [file formative_v3i4e15000_app1.docx]

**Use and acceptability of mobile phone surveys for noncommunicable disease behavioral risk surveillance administered using an interactive voice response questionnaire in rural Uganda: a qualitative study**

### IVR focused Focus group discussions guide

*These questions/topics are to be used as a guide for initiating and stimulating discussions during focus group discussions (FGD) with members of the community selected purposively to proxy for a similar demographic to be targeted in future mobile phone NCD risk behaviors surveys.*

*[Note to study team – this is a guide and the topics are to be used to check that the key aspects come out during the FGD, and for use to stimulate discussion as needed. They are not meant to be used as a questionnaire but rather to explore these issues in a conversational way. It is not necessary to use all the topics or questions. These are provided as an aide to ensure some key concepts are covered. Depending on time and knowledge of participants, the moderator can guide the discussion or leave to be free flowing if generally on topic. Be sure to record key points arising as well as non-verbal communication. After briefly introducing the project, please provide the FGD participants with instructions about how to access the IVR survey on their phones. All participants should have access to a mobile phone. In addition, please keep a record the types of mobile phones participants are using to complete the IVR survey].*

1. Aside from the IVR-delivered survey you took today, do you have any experiences (personal or in the community) with being asked questions in a health survey (regardless of topic)?
2. Aside from the IVR-delivered survey you took today, do you have any experiences (personal or in the community) with being asked survey questions related to NCD behavioural risk factors e.g. use of alcohol, food and fruit intake, physical inactivity, measuring of height, weight and blood pressure, etc. administered through mobile phone?
3. Aside from the IVR-delivered survey you took today, do you have any experiences (personal or in the community) with being asked questions in a health survey (regardless of topic) that was administered through mobile phone?
4. Aside from the IVR-delivered survey you took today, do you have any experiences (personal or in the community) with being asked survey questions related to NCD behavioural risk factors e.g. use of alcohol, food and fruit intake, physical inactivity, measuring of height, weight and blood pressure, etc. administered through mobile phone?
   1. Comment on your reaction on this experience or how you would react if you got such a survey
5. What were your overall impressions of the IVR-delivered survey that you just recently took? Was this your first time taking such a survey?
6. Did you have any frustrations in answering the IVR-delivered survey? [*Probe with question clarity, language, phrasing, audio quality, button response, and length if needed*]
7. Were there any aspects of the design, content, and functionality of the IVR-delivered survey that could be improved? Do you think most people from your community could complete a survey like the one you just took?
8. What did you particularly enjoy about the IVR-delivered survey?
9. What do you think the challenges would be to administer a survey like this within your community?
10. Do you have any recommendations to improve the likelihood that someone would respond to a survey and/or complete it?
    1. Specifically, is there a day in the week when people are more likely to respond to the IVR survey (because they will have more free time)?
    2. On that day, is there a specific time when people will more likely to answer the phone and take the survey?
11. Do you think it would be useful to send reminders to people before they are asked to complete the IVR survey?
    1. What type of reminder (text or SMS message, automated voice message) will be most useful?
    2. How many days before the IVR survey should the reminders be sent?
    3. Should multiple reminders be sent? If so, should we space out the reminders by a few days? Hours?
12. For people that receive an anonymous mobile phone survey, do you think the introduction has any determination in whether people answer the survey?
    1. How would you phrase a survey introduction?
    2. What key points are needed in the introduction?
    3. Before this group started, we asked you if the introduction’s narrator was a factor in answering a survey. Please give reasons for your answer.
13. Some of you indicated that you prefer a female voice and some prefer a male voice for the survey narration? What were some of the reasons for your choice? Do you think it would matter if you didn’t know you were receiving an IVR-delivered survey?
